# Identification of a Novel Immune-Related CpG Methylation Signature to Predict Prognosis in Stage II/III Colorectal Cancer

**DOI:** 10.3389/fgene.2021.684349

**Published:** 2021-06-28

**Authors:** Feng Chen, Lijuan Pei, Siyao Liu, Yan Lin, Xinyin Han, Erhong Meng, Xintong Wang, Shuai Hong, Dongliang Wang, Feide Liu, Yang Fei, Guangda Wang

**Affiliations:** ^1^Department of General Surgery, The Fourth Medical Center of PLA General Hospital, Beijing, China; ^2^ChosenMed Technology Co., Ltd., Beijing, China; ^3^Library, The Fourth Hospital of Hebei Medical University, Shijiazhuang, China; ^4^Computer Network Information Center, Chinese Academy of Sciences, Beijing, China; ^5^University of the Chinese Academy of Sciences, Beijing, China; ^6^Department of Radiology, The Fourth Hospital of Hebei Medical University, Shijiazhuang, China

**Keywords:** colorectal cancer, CpG methylated sites, biomarker, prognosis, immunotherapy

## Abstract

With the increasing incidence of colorectal cancer (CRC) and continued difficulty in treating it using immunotherapy, there is an urgent need to identify an effective immune-related biomarker associated with the survival and prognosis of patients with this disease. DNA methylation plays an essential role in maintaining cellular function, and changes in methylation patterns may contribute to the development of autoimmunity, aging, and cancer. In this study, we aimed to identify a novel immune-related methylated signature to aid in predicting the prognosis of patients with CRC. We investigated DNA methylation patterns in patients with stage II/III CRC using datasets from The cancer genome atlas (TCGA). Overall, 182 patients were randomly divided into training (*n* = 127) and test groups (*n* = 55). In the training group, five immune-related methylated CG sites (cg11621464, cg13565656, cg18976437, cg20505223, and cg20528583) were identified, and CG site-based risk scores were calculated using univariate Cox proportional hazards regression in patients with stage II/III CRC. Multivariate Cox regression analysis indicated that methylated signature was independent of other clinical parameters. The Kaplan–Meier analysis results showed that CG site-based risk scores could significantly help distinguish between high- and low-risk patients in both the training (*P* = 0.000296) and test groups (*P* = 0.022). The area under the receiver operating characteristic curve in the training and test groups were estimated to be 0.771 and 0.724, respectively, for prognosis prediction. Finally, stratified analysis results suggested the remarkable prognostic value of CG site-based risk scores in CRC subtypes. We identified five methylated CG sites that could be used as an efficient overall survival (OS)-related biomarker for stage II/III CRC patients.

## Introduction

In China, colorectal cancer (CRC) is the fifth most common malignancy, and CRC-related deaths have increased in recent years (Chen, 2015; Fang et al., 2015). Approximately 70% of patients with CRC have stage II/III tumors. At present, the tumor-node-metastasis classification criteria are insufficient to predict prognosis and make clinical decisions, especially in patients with stage II/III CRC (Edge and Compton, 2010). Considerable progress has been made in tumor immunotherapy (immuno-oncology) owing to the enhanced understanding of immune mechanisms. However, the benefit of immunotherapy in patients with CRC is limited, and the advancement in clinical research is relatively lagging (Sun et al., 2016). Programmed cell death protein 1/programmed death-1 ligand 1 antibody inhibitors have been reported to be ineffective in immunotherapy for 85% of patients with microsatellite stable (MSS) CRC (Sillo et al., 2019). In addition, existing biomarkers, including programmed death-1 ligand 1 protein expression, tumor mutational burden (TMB), immune scores, and gamma-interferon signatures, do not effectively predict the prognosis of patients with MSS CRC. Consequently, there is an urgent need to identify immune-related biomarkers for predicting cancer prognosis, which will improve the treatment of CRC.

Aberrant DNA methylation results in the downregulation of various genes and can potentially initiate the pathogenesis of cancer. It is a promising candidate for the development of robust diagnostic, predictive, and prognostic biomarkers for cancer. For instance, hypomethylation of long interspersed nuclear element-1 is correlated with poor survival in CRC patients (Antelo et al., 2012; Rhee et al., 2012). Additionally, long interspersed nuclear element-1 hypomethylation of cell-free DNA is associated with disease progression in CRC (Nagai et al., 2017). Moreover, the hypermethylation level of cyclin-dependent kinase inhibitor 2A predicts recurrence, distant metastasis, and prognosis in patients with CRC (Shen et al., 2007; Kim et al., 2010). Interestingly, cyclin-dependent kinase inhibitor 2A hypermethylation is associated with the poor survival of patients with rectal cancer after surgery and adjuvant 5-fluorouracil chemotherapy (Simpson et al., 1999; Kim et al., 2010). The methylation states of helicase-like transcription factor and hyperplastic polyposis 1 are correlated with tumor aggressiveness, recurrence, and prognosis (Huang et al., 2007). However, only a few studies have focused on identifying immune-related methylated signatures for predicting the prognosis of patients with stage II/III CRC. Therefore, it is necessary to identify prognosis-related methylated biomarkers for this deadly disease.

In this study, we aimed to identify and validate a novel immune-related methylated site-based signature using CRC datasets from the cancer genome atlas (TCGA). Based on our results, we proposed a prognosis-related biomarker that is also effective for patients with CRC subtypes.

## Materials and Methods

### Patients

**FIGURE 1:**
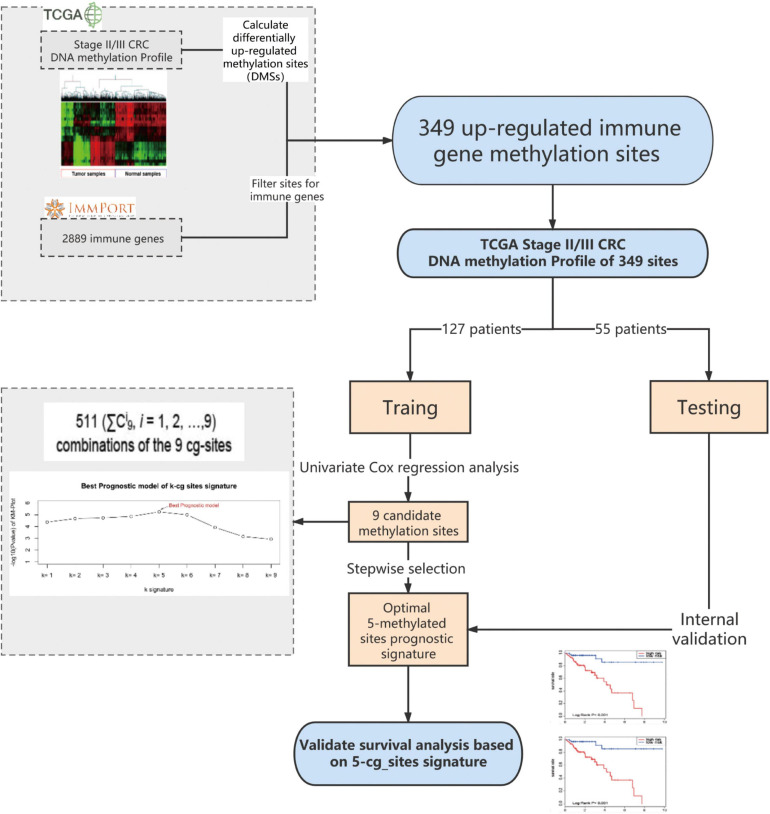
I Dentification of the methylated signature in the training set. Methylated site profiling in tumor and normal tissue samples. Overall, 349 methylated sites were overlapped between 6450 differentially methylated sites (DMSs) and 2483 immune genes. Correlation between nine methylated sites and the survival of patients with stage II/III CRC in the training group was observed upon performing Univariate Cox regression analysis. Development of a prognostic classifier for all combinations of the nine CG sites using the CG Score. For each combination, patients were classified into high- and low-risk groups based on their median CG Score, and the five-methylated site signature with the largest value of −log(p) was selected as the final signature. DMSs, differentially upregulated methylation sites; CRC, colorectal cancer; TCGA, the cancer genome atlas; CG score, CG site-based risk score.

We downloaded the epigenome-wide DNA CpG site methylation scored as a β-value between 0 and 1 (Illumina 450 K Methylation Beadchip) of stage II/III CRC samples from the Genomic Data Commons data portal^1^ (Sanford et al., 2018). Overall, 182 stage II/III CRC samples and 36 paired normal samples were included. The summary of patients is shown in Table 1, and the patients were randomly divided into training (*n* = 127) and testing groups (*n* = 55) (Figure 1). We obtained the fragments per kilobase of exon per million mapped fragment formats of 182 stage II/III CRC samples in the “HTSeq-FPKM” category, which were further processed, followed by normalized values for gene expression levels (Robinson et al., 2010; Kruppa and Jung, 2017). The “Masked Somatic Mutation” category included four types of mutation data based on diverse processing software, and we selected “MuTect2 Variant” process with 182 stage II/III CRC samples for further mutation analysis. TMB was determined by analyzing the number of somatic mutations per megabase. The cut-off value for high TMB (TMB-H) was determined to be the top 25% of all CRC patients. We obtained the clinical data of the 182 stage II/III CRC samples from TCGA-COAD dataset. The CRC samples gene expression profiles of GSE14333 and GSE103479 were downloaded from GEO databases^2^. The expression data of GSE14333 was based on GPL570 Platforms included 290 primary CRC samples (Submission date: January 08, 2010). The expression data of GSE103479 was based on GPL23985 Platforms included 363 stage II/III CRC samples (Submission date: December 31, 2017).

**TABLE 1:** Summary of patient demographics and characteristics.

**Characteristic**	**Training (*N* = 127)**	**Test (*N* = 55)**
**Gender**		
Female	58 (45.7%)	26 (47.3%)
Male	69 (54.3%)	29 (52.7%)
**Age**		
<65 years	50 (39.4%)	28 (50.9%)
≥65 years	77 (60.6%)	27 (49.1%)
**Stage**		
II	73 (57.5%)	32 (58.2%)
III	54 (42.5%)	23 (41.8%)
**Adjuvant chemotherapy**		
Adjuvant chemotherapy	49 (38.6%)	25 (45.5%)
None	78 (61.4%)	30 (54.5%)
**Vital status**		
Living	97 (76.4 %)	44 (80.0%)
Dead	30 (23.6%)	11 (20.0%)

### Identification of Immune-Related Differential Methylation Sites

First, 6450 differentially methylated sites (DMSs) between stage II/III CRC and adjacent normal tissues were identified using the edgeR package, with | log2 FC| > 1.0 and adjusted *P* < 0.05 as thresholds. Thereafter, we focused on the upregulated methylated sites between CRC and adjacent normal tissues and mapped them to immune genes. Overall, 2483 immune genes were downloaded from the immunology database and analysis portal (ImmPort)^3^. Finally, through this analysis, we identified 349 upregulated immune-related methylated sites in stage II/III CRC samples (Figure 1).

### Statistical Analysis

Machine learning algorithms for predictive models have been described previously (Hu et al., 2019). First, we used univariate and multivariate Cox proportional hazards regression to evaluate the association between overall survival (OS) and the methylation value of each gene site in the training group (Guo et al., 2018). Nine candidate CG sites associated with OS were screened (*P* < 0.1). We used a stepwise selection algorithm for selecting signatures to construct a reliable and an efficient predictive prognostic model (Figure 1). There are 511 (ΣC^*i*^_9_, *i* = 1, 2, …,9) combinations of the nine CG sites. For each combination, the CG site-based risk score (CG Score) was calculated based on the following equation, where N is the number of methylated sites of the signature, Meth_i_ is the methylation value of the candidate sites, and Coef_i_ is the univariate Cox regression coefficient:

(1) $$\mathrm{CG}\mathrm{Score}=\sum_{i=1}^{\text{N}}{\text{Meth}}_{\text{i}}*{\text{Coef}}_{\text{i}}$$

We calculated the CG Score for each sample and the median CG Score in the training group was used as the cut-off value (cut-off = 0.67). Next, we divided all samples into high- and low-risk groups. The Kaplan-Meier survival method, as well as the log-rank test, was applied to compare the prognosis between two groups. In this study, we used area under the curve (AUC) as the performance measurement method for predictive models, which was plotted using the “survivalROC” R package, and all statistical tests were performed using R-3.6.3.

### Immune Cell Infiltration in CRC

We calculated relative percent of 22 immune cells in each sample by CIBERSORT which included gene expression of 22 leukocyte subtypes (Newman et al., 2015). Then we compared 22 immune cells infiltrates level between high- and low- risk group samples by Wilcoxon ranked-sum test.

## Results

### A Five-Methylated Site Signature Predicts the Survival of Patients in the Training Group

The training group, comprising the complete clinical data, was used to further explore the association of 349 methylated sites with prognosis. Survival times were included as dependent variables in univariate Cox proportional hazard regression analysis of the 349 methylated sites. Nine methylated sites were found to be markedly associated with OS (*P* < 0.1) (Figure 1). Next, stepwise regression analysis was employed to provide the most effective predictive prognostic model, we developed a five-methylated site signature by selecting the best classification results to construct the final prognostic model (Supplementary Figure 1). The CG Score combining the five CG sites (cg11621464, cg13565656, cg18976437, cg20505223, and cg20528583) was determined as follows:

$$\text{CG}\mathrm{Score}=\left(1.87\times {\text{meth}}_{\mathrm{cg11621464}}\right)+\left(1.11\times {\text{meth}}_{\mathrm{cg13565656}}\right)$$

$$+\left(-1.74\times {\text{meth}}_{\mathrm{cg18976437}}\right)+\left(2.40\times {\text{meth}}_{\mathrm{cg20505223}}\right)+$$

(2) $$\left(-1.97\times {\text{meth}}_{\mathrm{cg20528583}}\right)$$

### Confirmation of OS Based on the Methylated Signature in the Training and Test Groups

**FIGURE 2:**
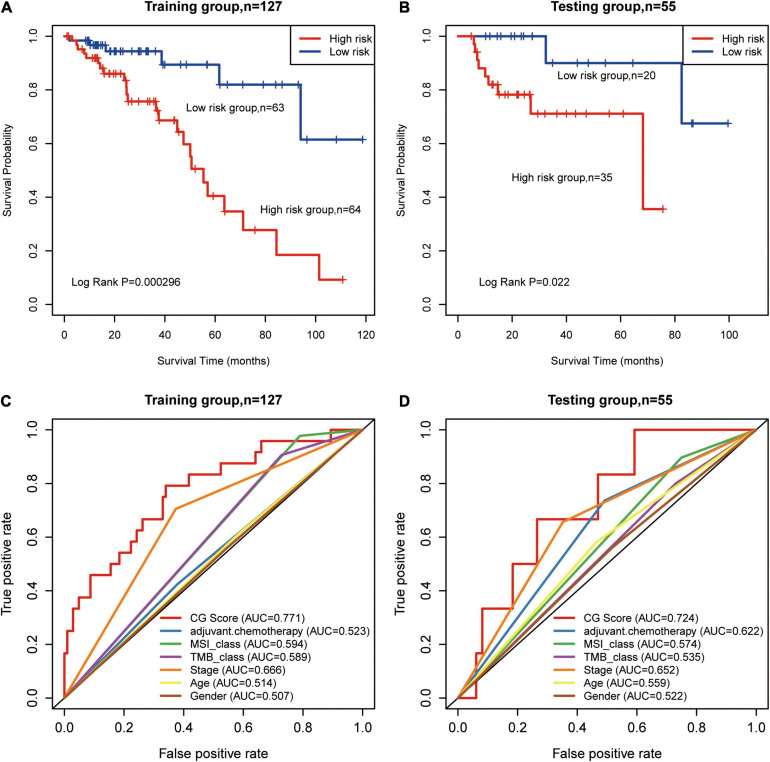
Prognosis of patients with stage II/III CRC was predicted using the methylated signature. **(A,B)** Based on Kaplan–Meier survival curves, patients with stage II/III CRC were classified into high- and low-risk groups using methylation sites as signature in the training and test groups. *P*-values were calculated via log-rank test. **(C,D)** Comparison of the sensitivity and specificity for the prediction of overall survival (OS) based on the CG Score and other clinical parameters. Receiver operating characteristics (ROC) curves for the **(C)** training and **(D)** test groups. CRC, colorectal cancer; MSI, microsatellite instability; TMB, tumor mutational burden; AUC, area under the curve; CG score, CG site-based risk score.

All patients in the training group were further divided into high- (*n* = 64) and low-risk groups (*n* = 63), and the OS in the low-risk group was higher than that in the high-risk group in the training group (HR: 3.18, 95% CI: 1.82–5.56; *P* = 0.000296, Figure 2A). Similarly, using the established prognostic model, patients in the test group were divided into high- (*n* = 35) and low-risk (*n* = 20) groups, and the OS in the low-risk group was higher than that in the high-risk group in the test group (HR: 1.75, 95% CI: 1.03–4.165; *P* = 0.022, Figure 2B). We calculated percent of 22 leukocyte cells of high- and low-risk groups by CIBERSORT and then compared immune cell fractions. As a result, we found high-risk group with more naive B cell (*p* < 0.05, Supplementary Figure 2B). Su et al. showed that after Chemotherapy-Induced Immunity, the B cells of patients with good curative effects were significantly reduced (Lu et al., 2020).

We used AUC to evaluate the accuracy of the prognostic model. In the training group, the predictive precision of the prognostic signature was more reliable than that of other clinical parameters (AUC_*CGScore*_ = 0.771, Figure 2C). Similar outcomes were obtained for the test group (AUC_*CGScore*_ = 0.724, Figure 2D). The decision curve analysis (DCA) curve showed that the diagnostic value of CG Score is due to clinical indicators, such as stage, age, etc., as well as existing immune biomarkers, such as microsatellite instability (MSI), TMB, etc. The combined model composed of these markers and CG Score can obtain a better net return rate ratio (Supplementary Figure 3). Therefore, our results suggest that CG Score may be an efficient prognostic biomarker.

### Methylated Signature Has Prognostic Value for CRC Subtypes

**FIGURE 3:**
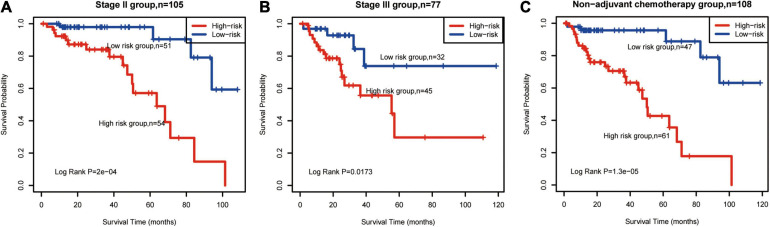
Survival prediction in patients with CRC subtypes. Kaplan–Meier survival curves classified patients into high- and low-risk groups using the methylated signature. **(A)** Stage II group (*n* = 105). **(B)** Stage III group (*n* = 77). **(C)**, non-adjuvant chemotherapy (*n* = 108). Vertical hash marks indicate censored data. CRC: colorectal cancer.

Overall, 182 CRC samples were classified into subtypes according to stage, TMB, MSI status, and adjuvant therapy. Next, we carried out a stratified analysis in subtypes to evaluate whether the methylated signature could predict the survival of patients within the same subtype. Log-rank tests of stage II (*P* = 0.0002, Figure 3A) and stage III patients (*P* = 0.0173, Figure 3B) showed that the methylated signature could classify stage II/III patients into high- and low-risk groups. The standard adjuvant therapy for patients with stage II/III CRC is oxaliplatin and fluorouracil chemotherapy for more than 6 months (Iveson et al., 2019). In the non-adjuvant chemotherapy subtypes, low-risk patients had significantly longer OS than high-risk patients (log-rank *P* = 1.3E-05, Figure 3C).

**FIGURE 4:**
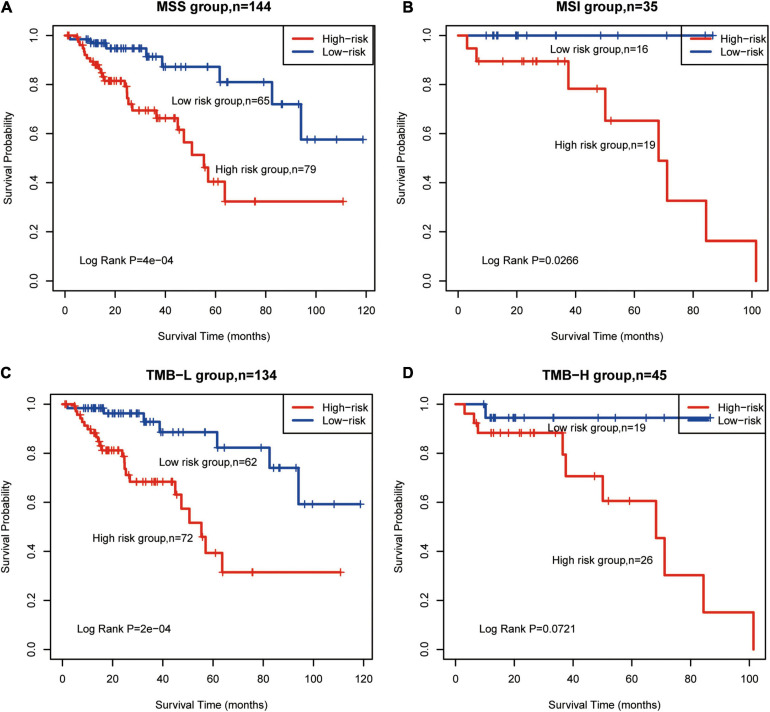
Survival prediction for TMB and MSI subtypes using the methylated signature. Based on Kaplan–Meier survival curves, patients with **(A)** MSS, **(B)** MSI, **(C)** TMB-L, and **(D)** TMB-H were classified into high- and low-risk groups using the methylated signature. Vertical hash marks indicate censored data. MSS, microsatellite stable; MSI, microsatellite instability; TMB-L, low tumor mutational burden; TMB-H, high tumor mutational burden.

TMB-H and MSI status are emerging biomarkers associated with immunotherapy for CRC (Schrock et al., 2019), but there were no significant differences estimated in OS between TMB and MSI subgroups (Supplementary Figure 4A,B). According to CG Score, low-risk MSS patients had significantly longer OS than high-risk patients (log-rank *P* = 0.0004, Figure 4A). This phenomenon was also identified in MSI (log-rank *P* = 0.0266, Figure 4B), TMB-L (log-rank *P* = 0.0002, Figure 4C) and TMB-H groups (log-rank *P* = 0.0721, Figure 4D). Thus, the results suggest that CG Score is an efficient prognostic tool for CRC subgroups.

### Methylated Signature Is an Independent Prognostic Factor

**FIGURE 5:**
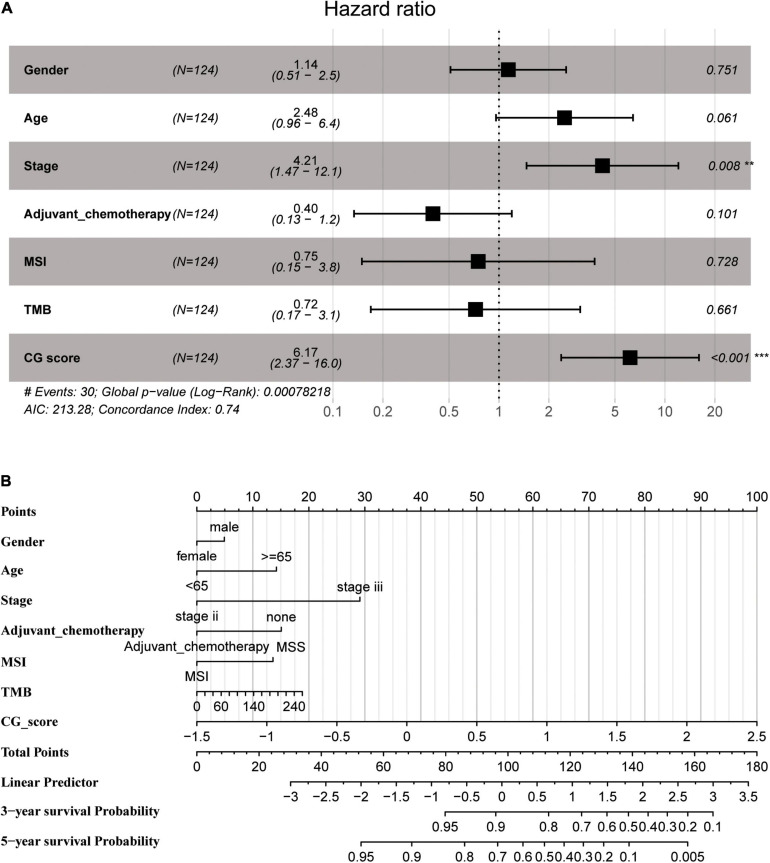
**(A)** Multivariate Cox regression analysis depicting the association of the methylated signature with the survival of stage II/III CRC patients in the training group. **(B)** The nomogram prediction model was developed by integrating CG Score with the clinical features in the training group. CRC, colorectal cancer; MSI, microsatellite instability; TMB, tumor mutational burden; CG score, CG site-based risk score.

A multivariate Cox regression analysis using CG Score and clinical parameters (e.g., age, sex, tumor stage, TMB, MSI status, and adjuvant chemotherapy) demonstrated that CG Score was independent of other clinical characteristics both in the training and test groups (Figure 5A and Supplementary Figure 5). In addition, the CG Score (HR: 6.17, 95% CI: 2.37–16.0, *P* < 0.001, *n* = 124, Figure 5A) could be a significant prognostic factor for patients in the high-risk group. According to the multivariate model contained clinicopathological information and the CG site-based risk score, we built a dynamic nomogram (Figure 5B).

### Correlations Between the Methylated Signature and Immune Biomarkers

Previous research showed immune checkpoint genes including PDL1, interferon-gamma (IFN-γ), PDL2, CTLA4, etc. We found negative correlations between CG Score and other markers (Supplementary Figure 6), which indicate the potential of CG Score to be a novel immune-related prognosis biomarker.

**FIGURE 6:**
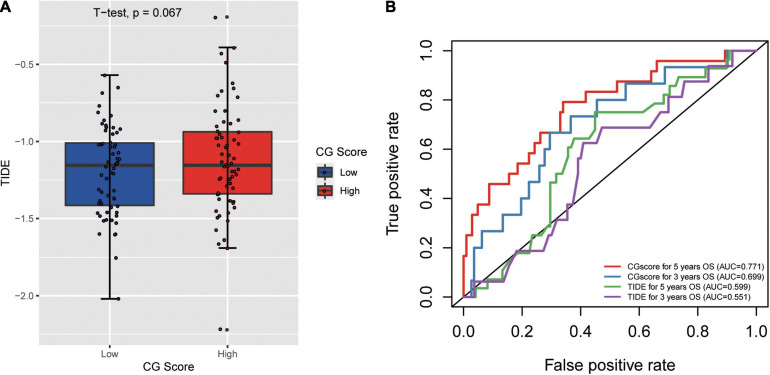
**(A)** Correlation between tumor mmune dysfunction and exclusion (TIDE) and CG Score. **(B)** The performance of the CG Score and TIDE for overall survival in CRC.

Tumor immune dysfunction and exclusion (TIDE) is a gene expression biomarker developed for predicting the clinical response to immune checkpoint blockade (Jiang et al., 2018). We obtained the TIDE score for 182 TCGA-CRC dataset by the online webserver^4^. There is different for TIDE score between high and low CG Score (*t*-test *p* = 0.067) and the AUC for CG Score under 5 and 3 years are 0.771 and 0.699. In addition, the AUC for TIDE under 5 and 3 years are 0.599 and 0.551 (Figure 6). The result indicated that CG Score might be a potential biomarker for immunotherapy especially for Immune checkpoint inhibitors, which show the better performance than existing signatures.

### RNA Expression Profile of the Methylated Signature

**FIGURE 7:**
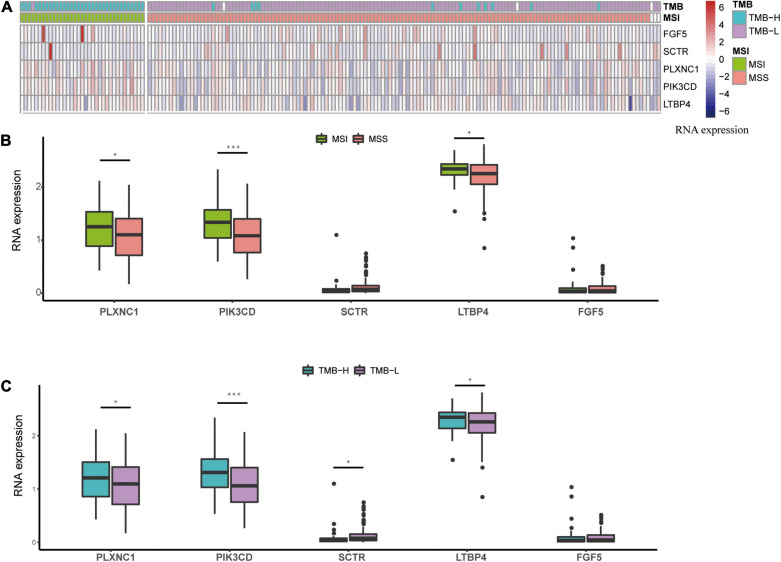
RNA expression profile based on the methylated signature. **(A)** Heatmap of expression levels, after z-score transformation, for the genes involved in the methylated signature. **(B)** The boxplot summarizes the mRNA expression levels in MSS and MSI samples. **(C)** The boxplot summarizes the mRNA expression levels in TMB-H and TMB-L samples. Asterisks indicate genes with significantly (*p* < 0.05) different expression as calculated by *t*-test. MSS: microsatellite stable; MSI: microsatellite instability; TMB-L, low tumor mutational burden; TMB-H, high tumor mutational burden.

The five methylated sites were identified on the following genes: *SCTR*, *PIK3CD*, *FGF5*, *PLXNC1*, and *LTBP4*. We observed that the high expression levels of *PIK3CD*, *PLXNC1*, and *LTBP4* were correlated with the MSI and TMB-H groups (Figure 7). Additionally, Chen J.-S. et al. (2019) found that *PIK3CD* was overexpressed in CRC. Li et al. (2019) confirmed that the overexpression of *PLXNC1* could promote cell proliferation and migration. According to a previous study, *LTBP4* acts as a local regulator of transforming growth factor-β expression during tissue deposition and signaling in CRC, and the increase in *LTBP4* expression might cause CRC (Berg et al., 2010). Our RNA expression profile analysis revealed that the above-mentioned genes could be related to MSI or TMB, and therefore, the signature has the potential to replace MSI or TMB as a new prognostic marker.

### External Validation of Signature in CRC Datasets

**FIGURE 8:**
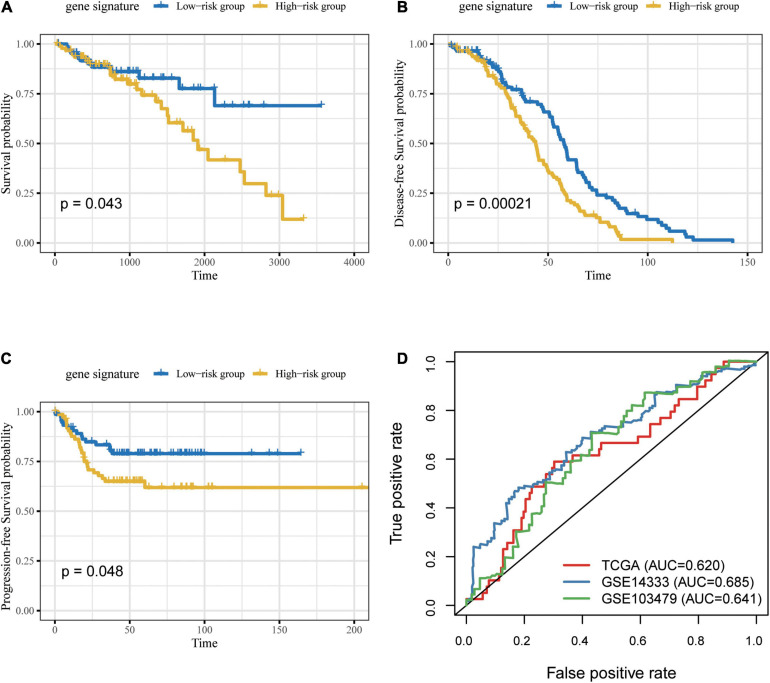
Prognosis of patients with CRC was predicted using the gene signature. Based on Kaplan–Meier survival curves, patients with CRC were classified into high- and low-risk groups using gene expression as signature in the **(A)** TCGA, **(B)** GSE14333 and **(C)** GSE103479 datasets. *P*-values were calculated via log-rank test. **(D)** Comparison of the sensitivity and specificity for the prediction of overall survival based on the gene-based CG Score. CRC, colorectal cancer; AUC, area under the curve.

Due to the incompleteness of the methylation profile with survival data or receiving ICB treatment of CRC patients, a large number of studies have confirmed that DNA methylation can cause changes in chromatin structure and DNA stability, thereby inhibiting gene expression (Huang et al., 2021) (Supplementary Figure 7). We built a gene model based on CG Score (New CG Score = CG Score^∗^correlation between gene expression and methylated sites) to assist in verifying the prognostic value of CG Score, and found that gene model can show good prognostic ability in independent verification datasets (log-rank *P*-value = 0.043, 0.00021, 0.048) (Figure 8).

## Discussion

A recent study reported that approximately 30% of CRC patients experience tumor recurrence in the first 3 years after surgery and adjuvant chemotherapy (Sargent et al., 2009). There is a close association between cancer recurrence and clinical or pathological characteristics, such as adjuvant chemotherapy and tumor-node-metastasis classification. However, due to tumor heterogeneity, patients harboring identical clinicopathological features or those undergoing therapeutic interventions present distinct relapse-free survival (Bathe and Farshidfar, 2014). In addition, MSI has become a highly effective immunotherapy biomarker for immune checkpoint inhibitors. About only 15% of stage II and III CRCs present a MSI or deficiency of DNA mismatch repair system (dMMR) phenotype, suggesting that associated with better prognosis than pMMR/MSS tumors (Sinicrope and Sargent, 2012). Moreover, patients with stage II/III dMMR/MSI CRC do not benefit from adjuvant fluoropyrimidine chemotherapy (Tougeron et al., 2016). Thus, it is necessary to propose a new molecular biomarker for predicting the prognosis of patients with stage II/III CRC, especially those with the MSS phenotype. Previous studies have reported that epigenetic modifications play a critical role in carcinogenesis. Promising outcomes have been observed with epigenetic drugs for the treatment of colon cancers (Raynal et al., 2016; Tan et al., 2019). However, it remains unclear whether epigenetic signatures can act as prognostic factors for CRC.

We employed various statistical methods to explore the relationship between methylated signature and prognosis in stage II/III CRC patients, and high-risk patients showed shorter OS than low-risk patients. The AUC for CG Score was estimated to be 0.771 and 0.724 in the training and test groups, respectively. GSEA analysis found that the most significant enrichment pathway in the low- risk group is the cell adhesion pathway, which the results further suggest that process of tumor invasion. Maurer’s study found that compared with normal tissues adjacent to cancer, the expression of ICAM-1 (intercellular adhesion molecular-1) in CRC tissues was significantly increased and positively correlated with the infiltration of inflammatory cells in the tumor microenvironment. The results of *in vitro* culture experiments show that the high expression of ICAM-1 depends on the increased dose of IFN-γ and IL-1β(Maurer et al., 1998; Xiang et al., 2001), For the high-risk cohort: KEGG analysis shows that the neuroactive ligand receptor interaction is mainly signaling pathway, that consistent with recent research (Yu et al., 2021) (Supplementary Figure 8). Moreover, CG Score was identified as an independent prognosis predictor for patients with CRC. We further discovered that CG Score could distinguish the prognosis of patients in the MSS, TMB-L, and TMB-H subgroups.

The five genes which methylated signature corresponded to after annotation, included *SCTR*, *PIK3CD*, *FGF5*, *PLXNC1*, and *LTBP4*. The hypermethylation of SCTR is a biomarker for precursor lesions in CRC detection (Chen J. et al., 2019; Li et al., 2020). Chen J.-S. et al. (2019) showed that PIK3CD induces CRC cell growth, migration, and invasion by activating AKT/GSK-3β/β-catenin signaling, suggesting that *PIK3CD* could be a novel prognostic biomarker and potential therapeutic target for CRC. Recent studies have shown that the methylated *FGF5* gene could potentially be used as a blood-based biomarker for detecting CRC (Mitchell et al., 2014). *PLXNC1* is involved in intracellular transport, cell migration, and activation of epidermal growth factor receptor and SMAD pathways (Ram et al., 2017). LTBP4 acts as a structural component of the extracellular matrix and local regulator of transforming growth factor-β during tissue deposition and signaling in CRC (Sterner-Kock et al., 2002). Based on the above-mentioned findings, all genes of the methylated signature play critical roles in the tumorigenesis and drug therapy of CRC. To date, only a few studies have investigated the prediction of prognosis in stage II/III CRC patients at the epigenomic level. Thus, in the present study, we proposed specific methylated sites for predicting the prognosis of patients with stage II/III CRC.

Although our CG Score can effectively aid in predicting the prognosis of CRC patients, there is a lack of clinical trials. In addition, there is no definitive evidence to show whether the five methylation sites we identified affect the usage of immune drugs. It will be more convincing if there are data to verify the efficacy of methylated signature and immunotherapy. Moreover, there is a need to verify the ability of the CG Score to distinguish the prognosis of stage I/IV CRC patients as well as the possibility of the CG Score in guiding the immune medication of these patients. In Genomics of Drug Sensitivity in Cancer (GDSC), there are no chemotherapeutics Drugs for the methylation signature. The most sensitive drugs targeting PI3K-Akt signaling pathway (PIK3CA-D) are Alpelisib and Taselisib. If future studies find a drug suitable for the CpG site, experiments can be conducted to verify the sensitivity of the drug.

## Data Availability Statement

The original contributions presented in the study are included in the article/Supplementary Material, further inquiries can be directed to the corresponding authors.

## Author Contributions

GW, YF, and FL conceived and designed the study. FC, LP, EM, and XW acquired the data and drafted the manuscript. SH, DW, YL, and XH analyzed and interpreted the data. XH and SL critically revised the manuscript for important intellectual content. SL, FL, GW, and YF approved the version of the manuscript to be published. All authors contributed to the manuscript and approved the submitted version.

## Conflict of Interest

SL, XW, EM, SH, and DW were employed by ChosenMed Technology (Beijing) Co., Ltd. The remaining authors declare that the research was conducted in the absence of any commercial or financial relationships that could be construed as a potential conflict of interest.

## Footnotes

1 https://portal.gdc.cancer.gov/

2 http://www.ncbi.nlm.nih.gov/geo/

3 https://www.immport.org/shared/genelists

4 http://tide.dfci.harvard.edu/

## Abbreviations

CRC: colorectal cancer
MSS: microsatellite stable
TMB: tumor mutational burden
TMB-H: high tumor mutational burden
OS: overall survival
CG score: CG site-based risk score
AUC: area under the curve
MSI: microsatellite instability
DCA: decision curve analysis.
