# Supplementary material for: Identification of a Novel Immune-Related CpG Methylation Signature to Predict Prognosis in Stage II/III Colorectal Cancer
Source: Front Genet. 2021 Jun 28;12:684349. doi: 10.3389/fgene.2021.684349 (PMC8273301; doi:10.3389/fgene.2021.684349)
Supplement: Supplementary file 1 [file Data_Sheet_1.docx]

Supplementary Material

# Supplementary Figures and Tables

## Supplementary Figures


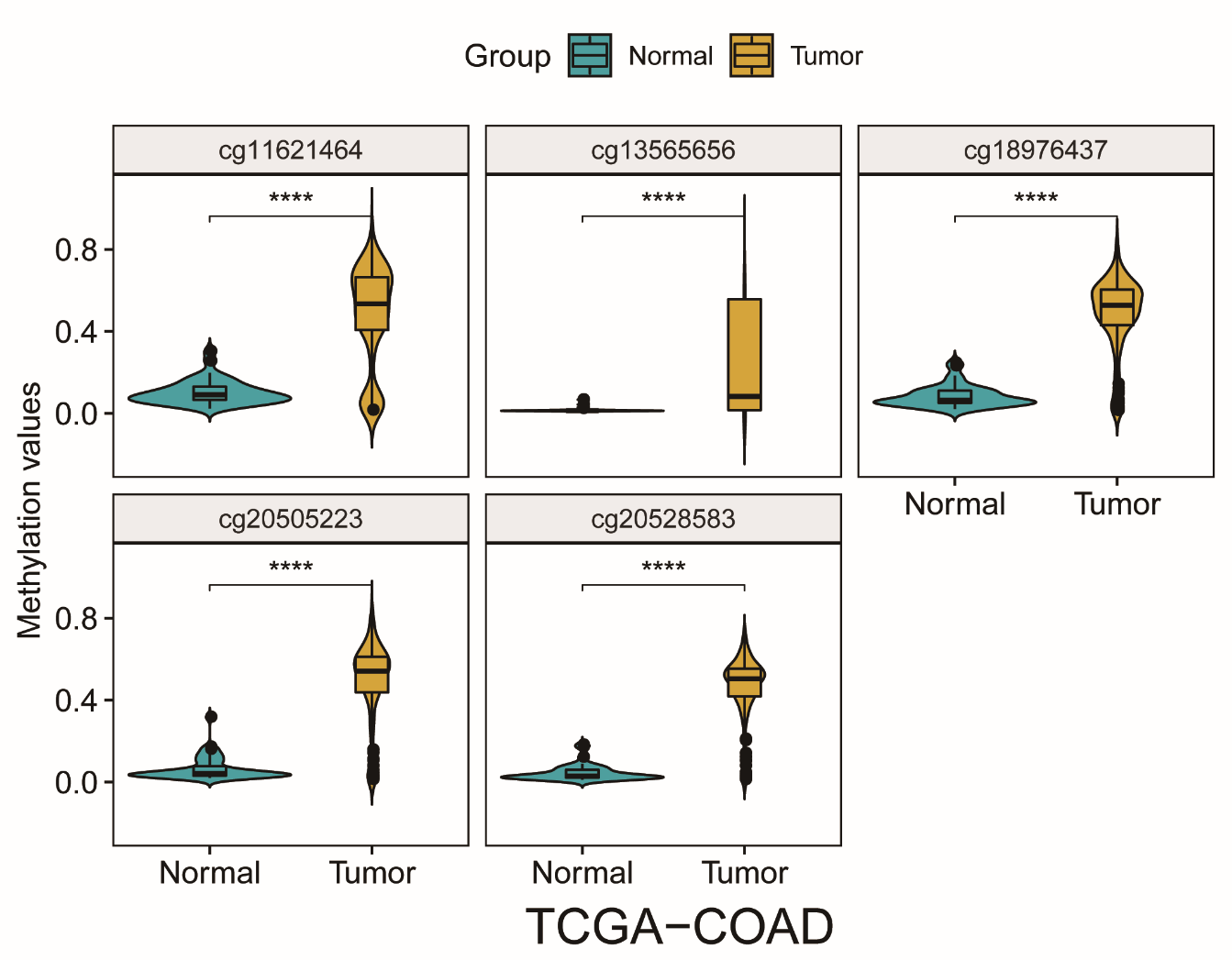


**Supplementary Figure 1.** The methylation values of sites from TCGA database. TCGA: The Cancer Genome Atlas; COAD: colon adenocarcinoma.


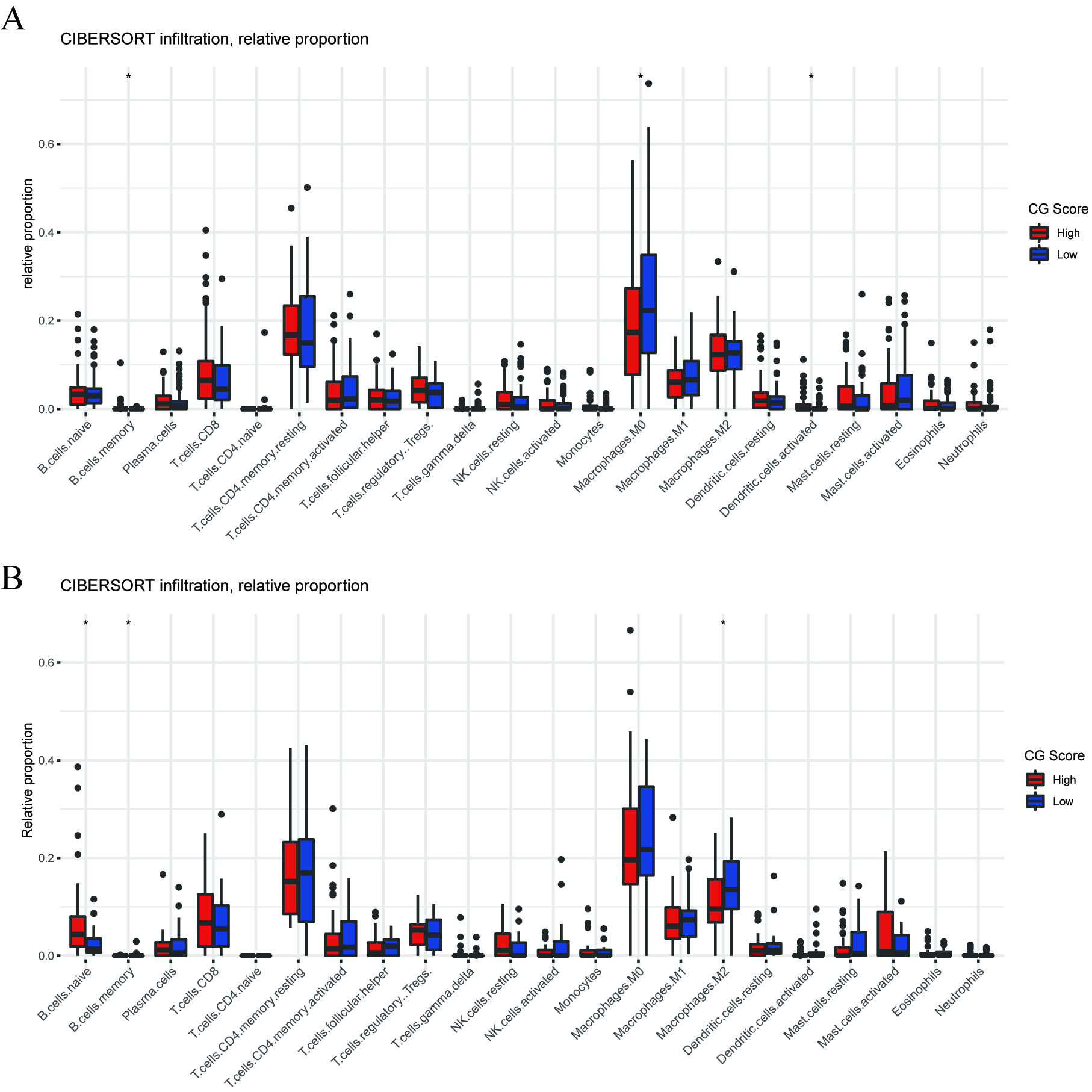


**Supplementary Figure 2.** Comparison of immune cell infiltration between between high- and low- risk group in (A)Training and (B)Test group.

**
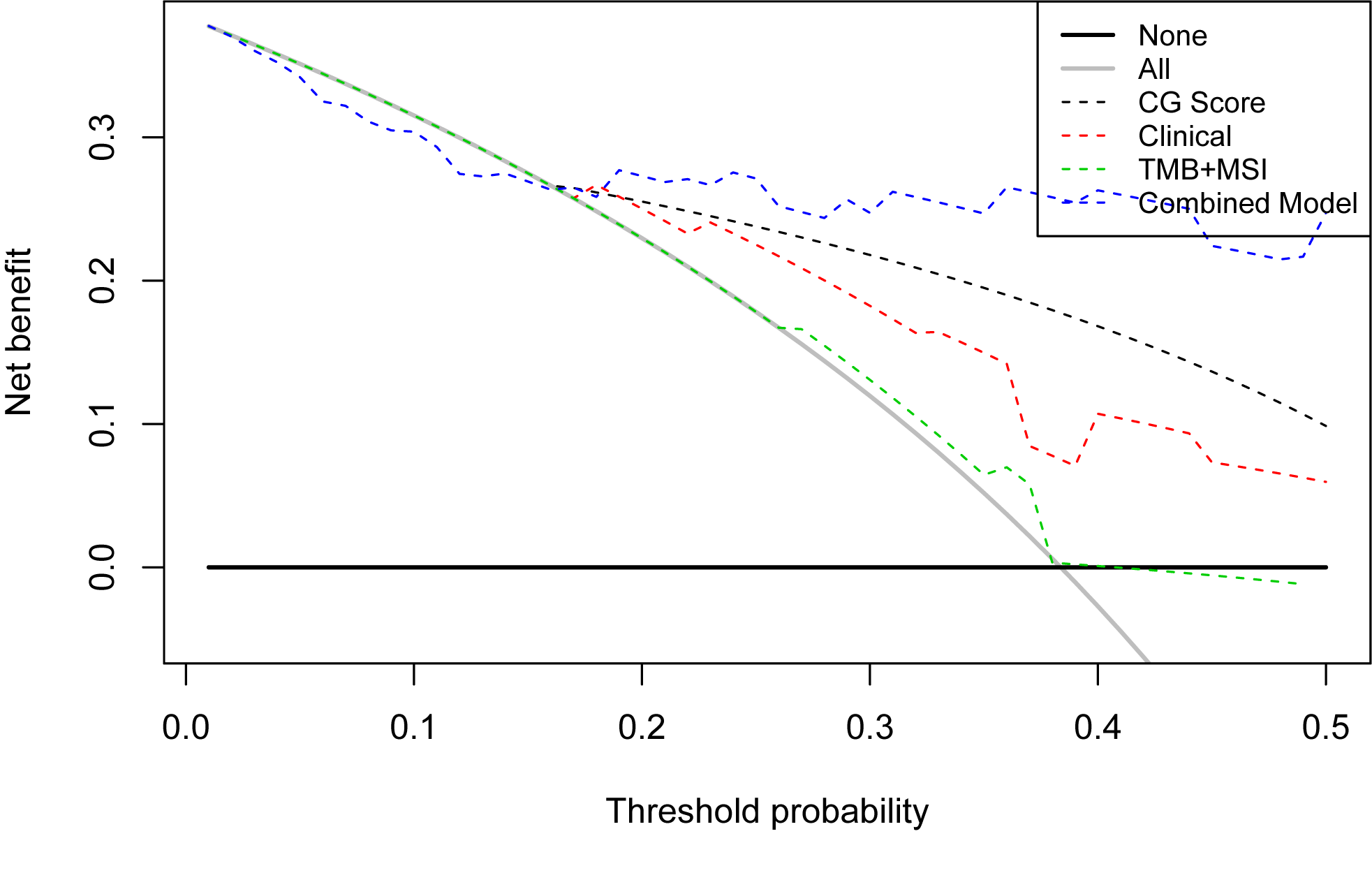
**

**Supplementary Figure 3.**  Decision curve analysis for CG Score ,clinical features, immune signatures and a combined model.


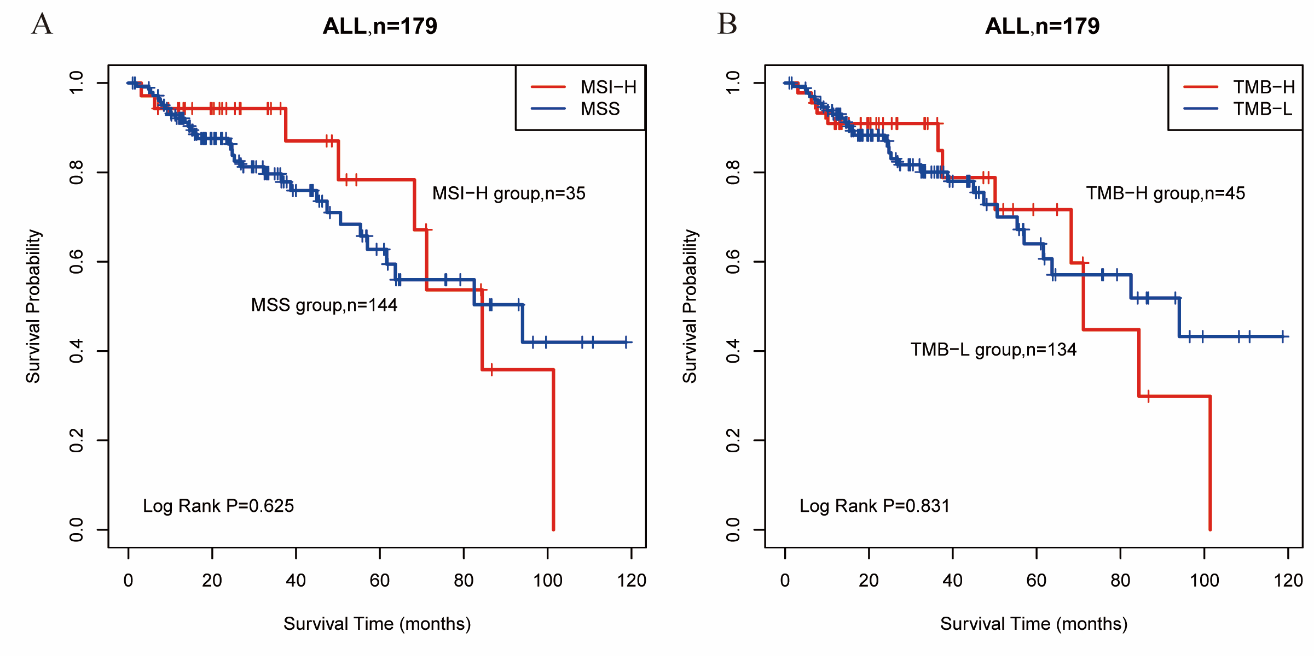


**Supplementary Figure 4.** Kaplan–Meier curve for TMB and MSI as prognostic markers. MSS: microsatellite stable; MSI-H: high microsatellite instability; TMB-L: low tumor mutational burden; TMB-H: high tumor mutational burden.


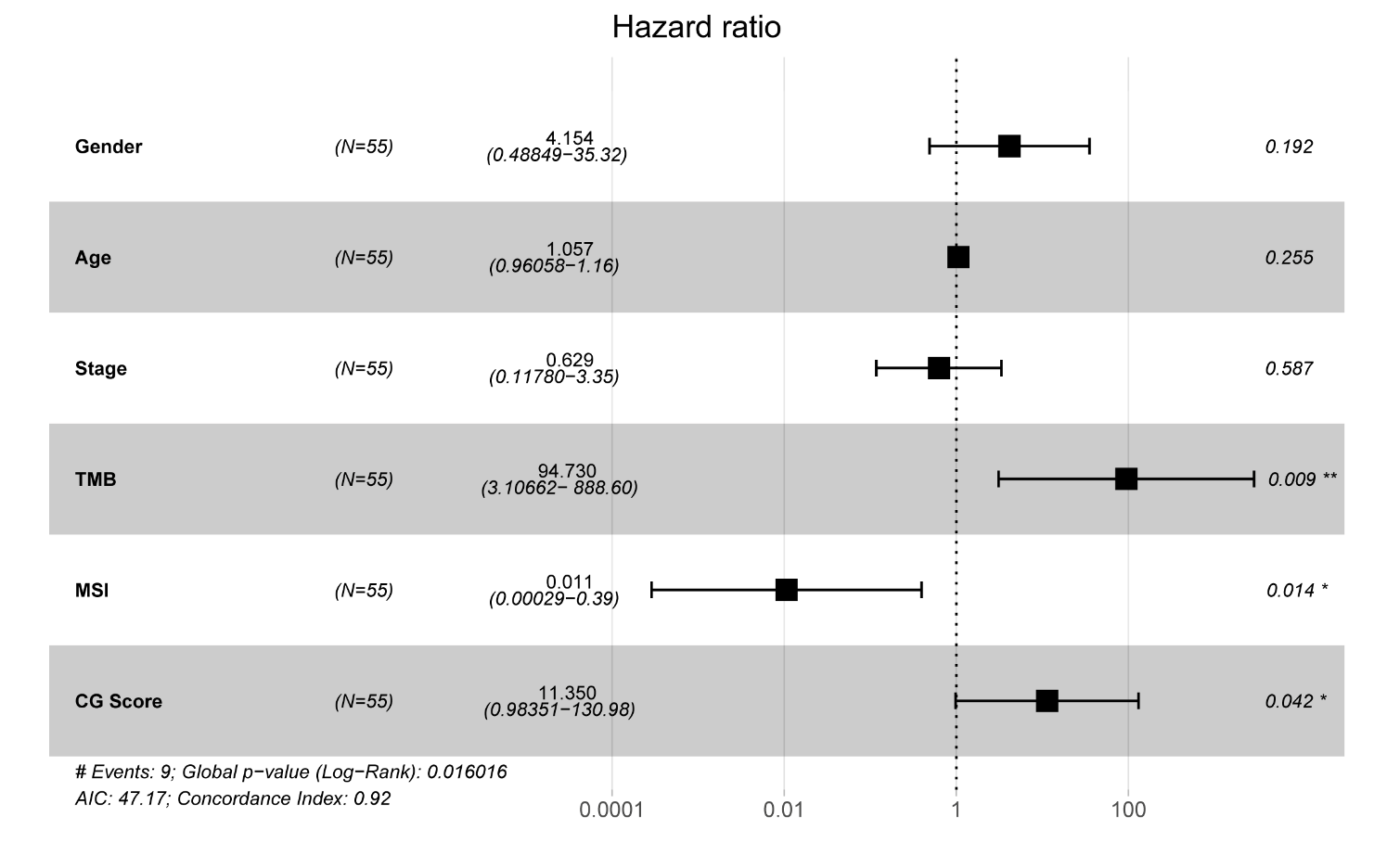


**Supplementary Figure 5.** Multivariate Cox regression analysis revealing the association between the signature and survival of patients with stage II/III CRC in the test group. CRC: colorectal cancer; MSI: microsatellite instability; TMB: tumor mutational burden; CG score: CG site-based risk score.


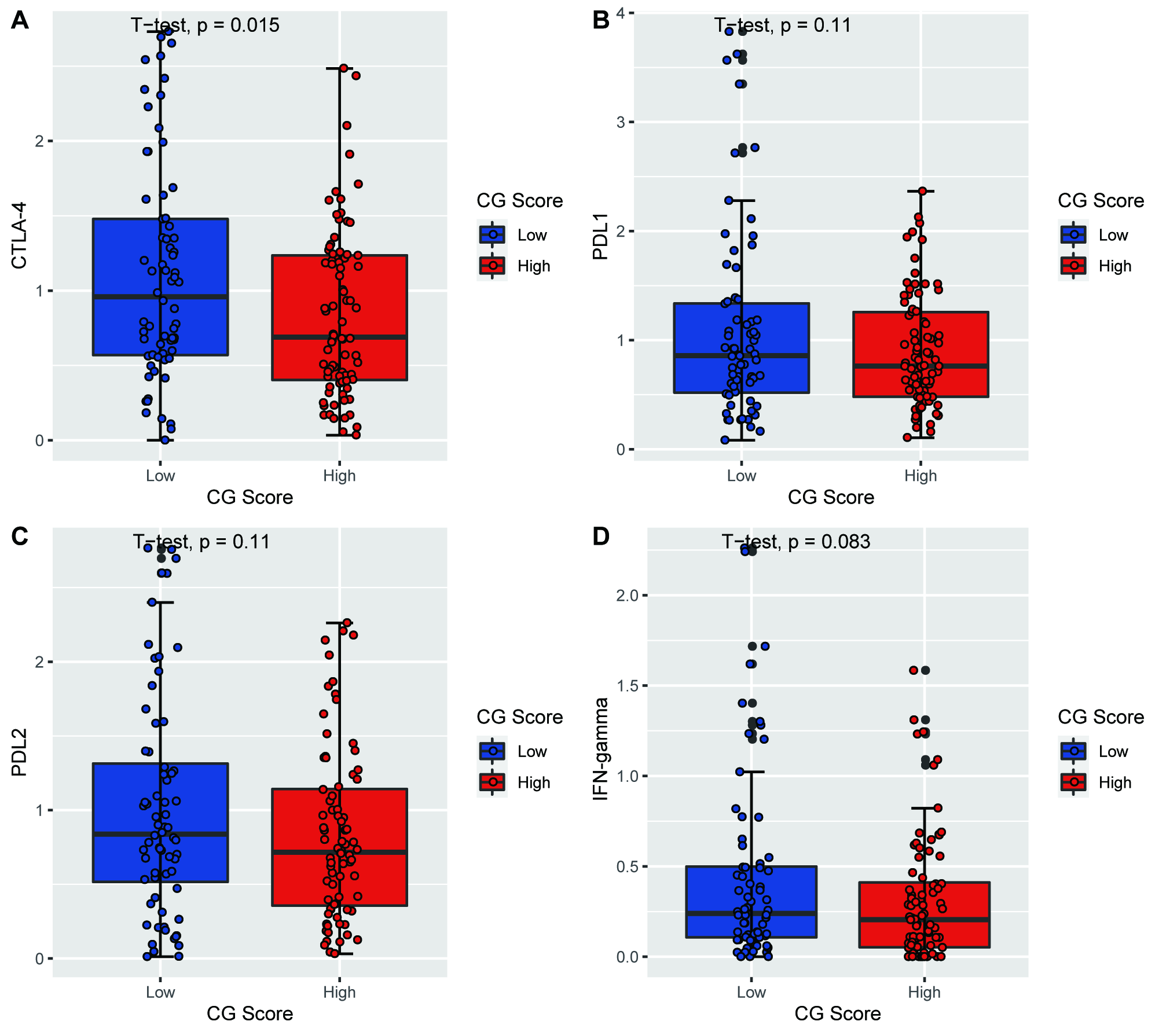


**Supplementary Figure 6.** Relationship between the expression of immune markers and CG Score.


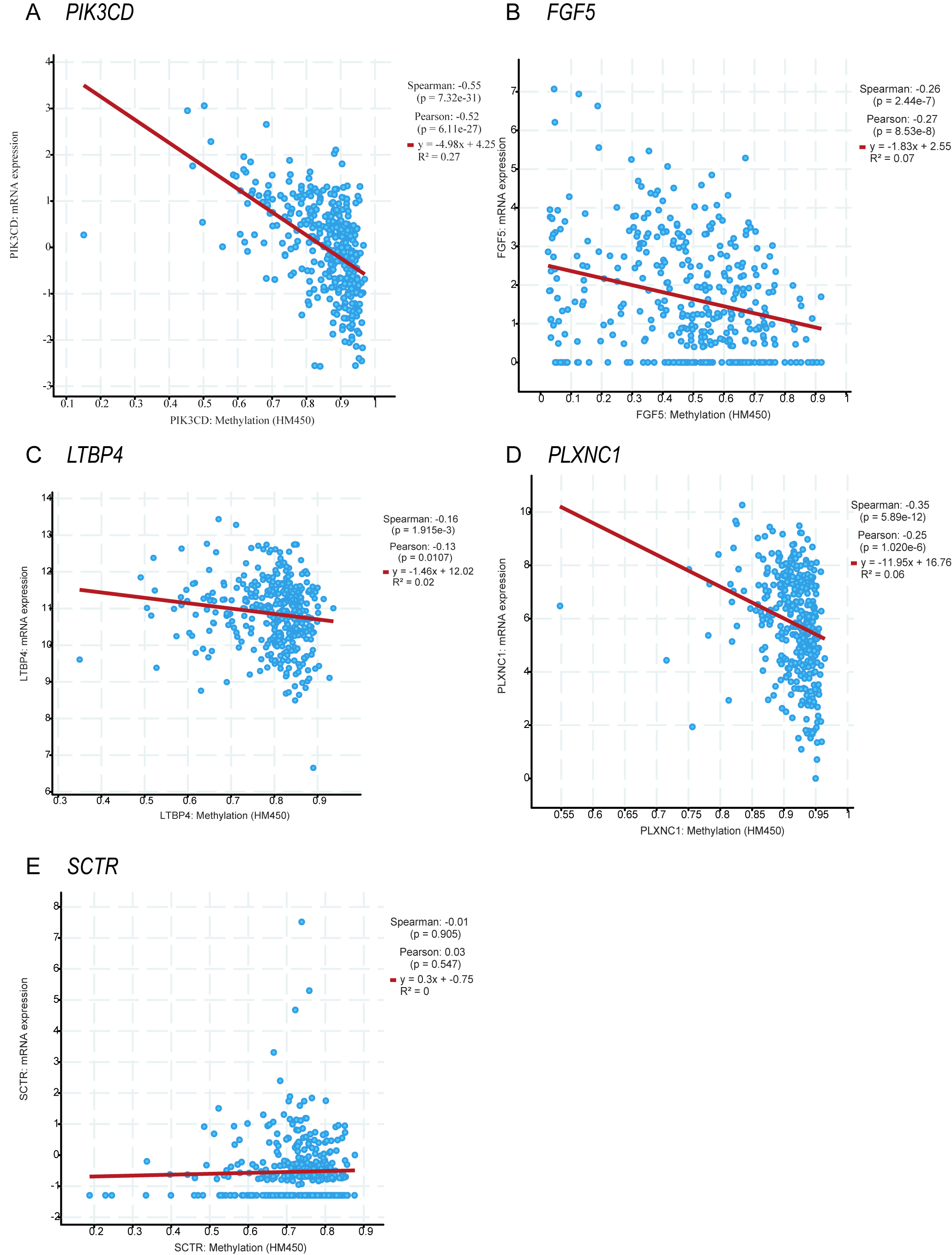


**Supplementary Figure 7.** Relationship between the expression of five gene and five methylation sites of CpG island. (A) PIK3CD, (B) FGF5, (C) LTBP4, (D) PLXNC1, (E) SCTR.


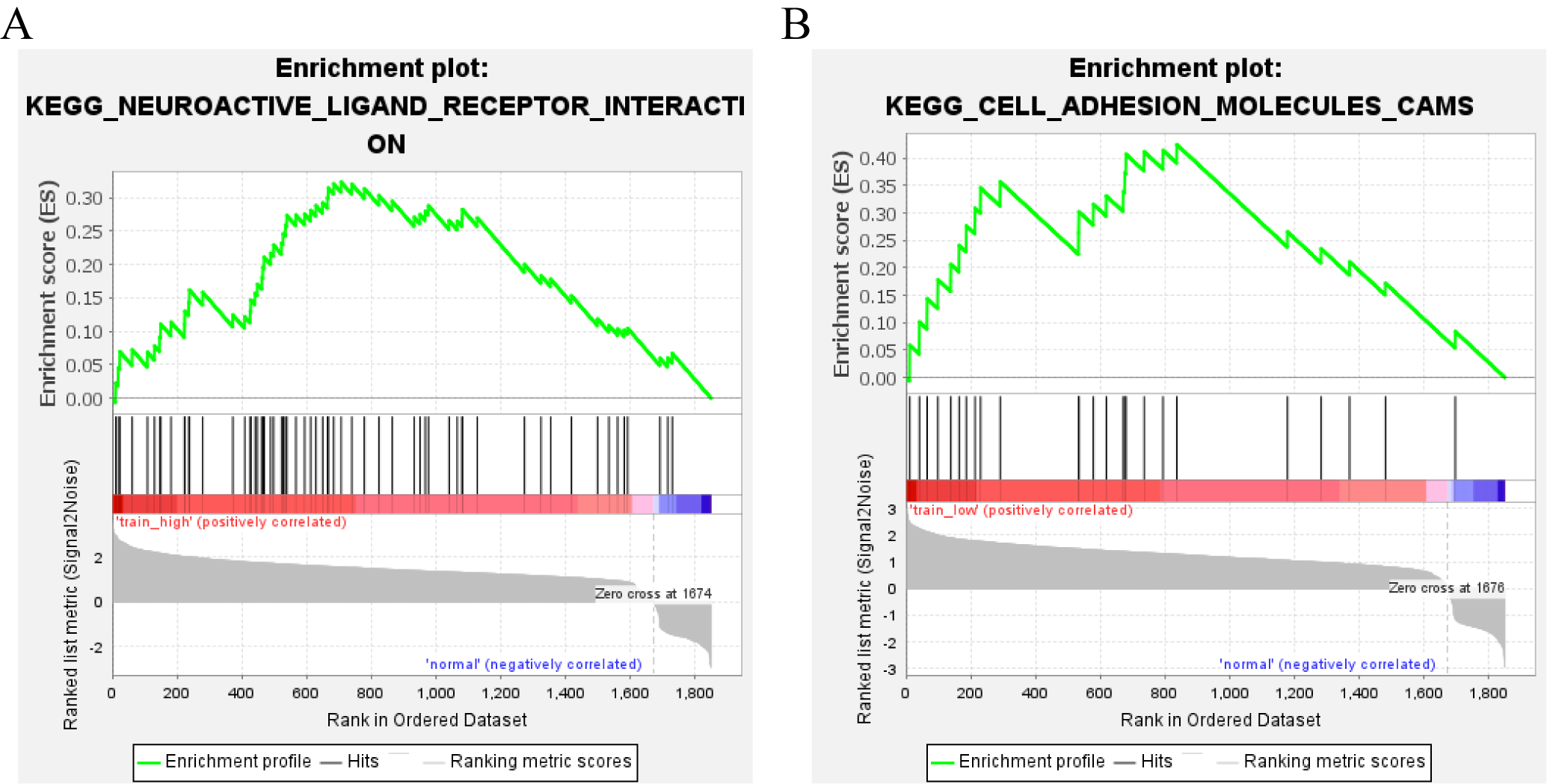


**Supplementary Figure 8**  GSEA analysis of KEGG pathways for differentially methylated sites in (A)high- and (B) low-risk groups.
